# Multistep endoscopic approach for recalcitrant colorectal anastomosis

**DOI:** 10.1055/a-2239-4662

**Published:** 2024-02-07

**Authors:** Jaime Escobar Ortiz, Jorge Pérez Pérez, José Carlos Villa Poza, Francisco Garrido, Marta Castillo, Ángel Ponferrada Díaz, Álvaro Martínez Alcalá García

**Affiliations:** 1 Aparato Digestivo, Hospital Universitario Infanta Leonor, Madrid, Spain; 2 Endoscopy, Centro de Innovaciones Digestivas Martinez Alcalá, Seville, Spain; 3 Endoscopia digestiva, Hospital Universitario Infanta Leonor, Madrid, Spain

We present the case of a 69-year-old man with a history of sigmoid colon adenocarcinoma pN1c treated by laparoscopic sigmoidectomy who required a Hartmann’s intervention for anastomotic dehiscence. The patient subsequently underwent reconstruction of the colon by terminolateral anastomosis. However, he then developed a symptomatic stenosis in the region of the colorectal anastomosis, which resulted in recurrent subocclusive episodes.

The first approach taken to treat the stenosis was endoscopic balloon dilation. Unfortunately, during the second dilation session (18 mm), an iatrogenic perforation occurred. This perforation was treated with hemoclips, without further complication ( Video 1 ).

Multistep endoscopic approach for recalcitrant colorectal anastomosis.Video 1

In the follow-up period after balloon dilation, the patient was admitted to the emergency room with a subocclusive episode. As salvage therapy to avoid Hartmann’s intervention, we decided to insert a luminal apposition stent (16 × 20 mm), as described in the literature 1 2 . The stent was removed 8 weeks later without any secondary events ( Video 1 ).

At 8 weeks after removal of the prosthesis, the patient presented with mild symptoms of abdominal pain, increased constipation, and increased need for laxative treatment. A new rectoscopy revealed a millimetric re-stenosis of the anastomosis with keloid appearance ( Fig. 1 **a** ). A new approach was therefore adopted, involving endoscopic stricturoplasty using an ITknife nano (Olympus, Tokyo, Japan), with injection of triamcinolone in four quadrants ( Fig. 1 **b** ). This technique has been previously described for esophagogastric stricture 3 . The patient underwent two more sessions of triamcinolone injection to reduce the keloid component.

**Fig. 1:**
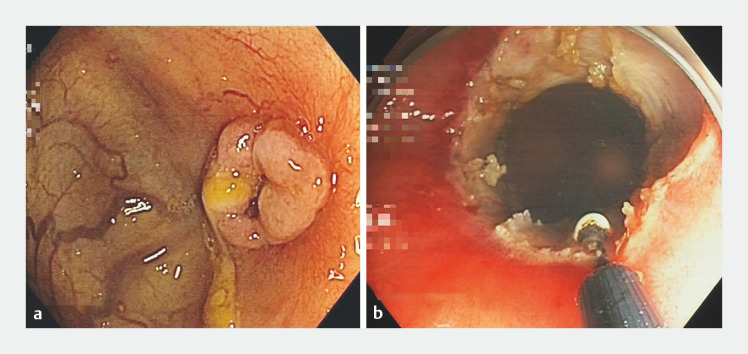
Endoscopic view. **a** The stenosis with keloid component before stricturoplasty. **b** The stenosis after completion of stricturoplasty.

During the subsequent follow-up period, the patient remained asymptomatic without recurrent stenosis.

This case demonstrates a complex postoperative colorectal stricture for which multiple step-up endoscopic treatments were mandatory to achieve luminal patency. An important take-home message of this case is that the failure of a given technique should not discourage further attempts at techniques aimed at restoring luminal patency, including endoscopic resection of the fibrotic ring.

Endoscopy_UCTN_Code_TTT_1AQ_2AF

## Notes

Endoscopy E-Videos https://eref.thieme.de/e-videos
*E-Videos* is an open access online section of the journal *Endoscopy* , reporting on interesting cases and new techniques in gastroenterological endoscopy. All papers include a high-quality video and are published with a Creative Commons CC-BY license. Endoscopy E-Videos qualify for HINARI discounts and waivers and eligibility is automatically checked during the submission process. We grant 100% waivers to articles whose corresponding authors are based in Group A countries and 50% waivers to those who are based in Group B countries as classified by Research4Life (see: https://www.research4life.org/access/eligibility/ ). This section has its own submission website at https://mc.manuscriptcentral.com/e-videos .
